# (3-Phenyl­isoxazol-5-yl)methanol

**DOI:** 10.1107/S1600536810003417

**Published:** 2010-02-06

**Authors:** Ling Yin, Gui-Long Zhao, Jiong Jia, Qing-Yang Meng, Jian-wu Wang

**Affiliations:** a School of Chemistry and Chemical Engineering, Shandong University, Jinan 250100, People’s Republic of China; b Tianjin Key Laboratory of Molecular Design and Drug Discovery, Tianjin Institute of Pharmaceutical Research, Tianjin 300193, People’s Republic of China

## Abstract

In the title compound, C_10_H_9_NO_2_, the isoxazole and phenyl rings form a dihedral angle of 25.82 (3)°. In the crystal, inter­molecular O—H⋯O hydrogen bonds link the mol­ecules into ribbons propagating along [001]. The crystal packing is further stabilized by weak C—H⋯O and C—H⋯N inter­actions.

## Related literature

For related structures, see: Tian & Li (2006 ▶); Tang *et al.* (2006 ▶).
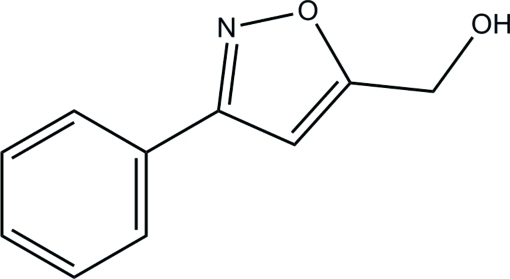


## Experimental

### 

#### Crystal data

C_10_H_9_NO_2_
*M* *_r_* = 175.18
Monoclinic, 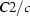

*a* = 41.03 (4) Å
*b* = 5.694 (5) Å
*c* = 7.348 (7) Å
β = 98.51 (2)°
*V* = 1698 (3) Å^3^
*Z* = 8
Mo *K*α radiation
μ = 0.10 mm^−1^
*T* = 298 K
0.55 × 0.45 × 0.02 mm

#### Data collection

Bruker SMART APEX diffractometer
3328 measured reflections
1500 independent reflections
1182 reflections with *I* > 2σ(*I*)
*R*_int_ = 0.059

#### Refinement

*R*[*F* ^2^ > 2σ(*F* ^2^)] = 0.054
*wR*(*F* ^2^) = 0.119
*S* = 1.05
1500 reflections
118 parameters
H-atom parameters constrained
Δρ_max_ = 0.16 e Å^−3^
Δρ_min_ = −0.23 e Å^−3^

### 

Data collection: *SMART* (Bruker, 1998 ▶); cell refinement: *SAINT* (Bruker, 1999 ▶); data reduction: *SAINT*; program(s) used to solve structure: *SHELXTL* (Sheldrick, 2008 ▶); program(s) used to refine structure: *SHELXTL*; molecular graphics: *SHELXTL*; software used to prepare material for publication: *SHELXTL*.

## Supplementary Material

Crystal structure: contains datablocks I, global. DOI: 10.1107/S1600536810003417/cv2692sup1.cif

Structure factors: contains datablocks I. DOI: 10.1107/S1600536810003417/cv2692Isup2.hkl

Additional supplementary materials:  crystallographic information; 3D view; checkCIF report

## Figures and Tables

**Table 1:** Hydrogen-bond geometry (Å, °)

*D*—H⋯*A*	*D*—H	H⋯*A*	*D*⋯*A*	*D*—H⋯*A*
O2—H2*B*⋯O2^i^	0.86	1.89	2.669 (4)	151
O2—H2*A*⋯O2^ii^	0.88	2.09	2.677 (5)	124
C8—H8⋯N1^iii^	0.93	2.61	3.542 (4)	177
C10—H10*B*⋯O2^iv^	0.97	2.58	3.352 (4)	137

Symmetry codes: (i) 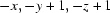; (ii) 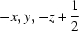; (iii) 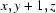; (iv) 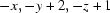.

**Fig. 1.**
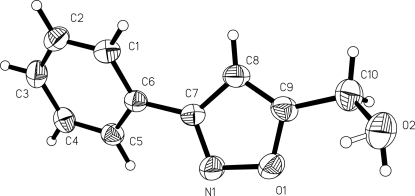
The molecular structure of (I), with atom labels and 40% probability displacement ellipsoids for non-H atoms.

## supplementary crystallographic information

### Comment

In continuation of our study of isoxazole derivatives (Tang *et al.*, 2006), we present here the crystal structure of the title compound, (I).

In (I) (Fig. 1), the bond lengths and angles of the isoxazole ring are normal and comparable to those reported for related structures (Tian & Li, 2006; Tang *et al.*, 2006). The mean planes of the benzene (C1—C6) and isoxazole (C7—C9/N1/O1) rings make a dihedral angle of 25.82 (3)°. Intermolecular O—H···O hydrogen bonds (Table 1) link the molecules into ribbons extended along the c axis. The crystal packing is further stabilized by weak C—H···O and C—H···N interactions (Table 1).

### Experimental

A round-bottomed flask was charged with benzaldoxime(5.0 g, 41.3 mmol) in 20 ml N, *N*-dimethyl formamide, and then *N*-chlorosuccinimide (1.20 g, 9.1 mmol) was added to the solution. Heating until *N*-chlorosuccinimide was solved, and the mixture was stirred at room temperature for 20 min, into the above solution was added *N*-chlorosuccinimide (4.80 g, 36.4 mmol) in batches under 308 K. After 3 h, propargyl alcohol ( 2.78 g, 49.6 mmol) was added, then the saturated solution of CuSO4.5H2O (0.62 g, 2.48 mmol) and L-ascorbic acid (1.75 g, 9.92 mmol) were added also. The solution of K_2_CO_3_ (3.14 g, 45.5 mmol) was added to the mixture and was stirred for 1 h. The reaction mixture was diluted with the saturated solution of ethylenediaminetetraacetic acid, and then was extracted with dichloromethane (3 times per 50 ml), and the extracts were dried over anhydrous Na_2_SO_4_. The solution was evaporated to afford a residue, which was purified by a silica-gel column chromatography (petroleum ether / ethyl acetate, 3:1 by volume) to afford a pale yellow solid (yield: 61.7%, m.p. 325–326 K). The crystals of (I) were obtained from petroleum ether / ethyl acetate / dichloromethane (3:1:1, V/V/V) by slow evaporation at room temperature.

### Refinement

All H atoms were placed in calculated positions, with C—H = 0.93 or 0.97 Å, O—H = 0.86, 0.88 Å, and included in the final cycles of refinement using a riding model, with U_iso_(H) = 1.2-1.5U_eq_(C, O) The hydroxyl H atom was treated as disordered over two positions with equal occupancies fixed to 0.5.

### Crystal data

**Table:**

C_10_H_9_NO_2_	*F*(000) = 736
*M**_r_* = 175.18	*D*_x_ = 1.371 Mg m^−^^3^
Monoclinic, *C*2/*c*	Mo *K*α radiation, λ = 0.71073 Å
Hall symbol: -C 2yc	Cell parameters from 1357 reflections
*a* = 41.03 (4) Å	θ = 3.0–28.1°
*b* = 5.694 (5) Å	µ = 0.10 mm^−^^1^
*c* = 7.348 (7) Å	*T* = 298 K
β = 98.51 (2)°	Plate, colourless
*V* = 1698 (3) Å^3^	0.55 × 0.45 × 0.02 mm
*Z* = 8	

### Data collection

**Table:**

Bruker SMART APEX diffractometer	1182 reflections with *I* > 2σ(*I*)
Radiation source: fine-focus sealed tube	*R*_int_ = 0.059
graphite	θ_max_ = 25.1°, θ_min_ = 2.0°
phi and ω scans	*h* = −48→43
3328 measured reflections	*k* = −6→5
1500 independent reflections	*l* = −8→8

### Refinement

**Table:**

Refinement on *F*^2^	Primary atom site location: structure-invariant direct methods
Least-squares matrix: full	Secondary atom site location: difference Fourier map
*R*[*F*^2^ > 2σ(*F*^2^)] = 0.054	Hydrogen site location: inferred from neighbouring sites
*wR*(*F*^2^) = 0.119	H-atom parameters constrained
*S* = 1.05	*w* = 1/[σ^2^(*F*_o_^2^) + (0.0354*P*)^2^ + 0.7771*P*] where *P* = (*F*_o_^2^ + 2*F*_c_^2^)/3
1500 reflections	(Δ/σ)_max_ < 0.001
118 parameters	Δρ_max_ = 0.16 e Å^−^^3^
0 restraints	Δρ_min_ = −0.23 e Å^−^^3^

### Special details

**Table:**

Geometry. All esds (except the esd in the dihedral angle between two l.s. planes) are estimated using the full covariance matrix. The cell esds are taken into account individually in the estimation of esds in distances, angles and torsion angles; correlations between esds in cell parameters are only used when they are defined by crystal symmetry. An approximate (isotropic) treatment of cell esds is used for estimating esds involving l.s. planes.
Refinement. Refinement of *F*^2^ against ALL reflections. The weighted *R*-factor wR and goodness of fit *S* are based on *F*^2^, conventional *R*-factors *R* are based on *F*, with *F* set to zero for negative *F*^2^. The threshold expression of *F*^2^ > σ(*F*^2^) is used only for calculating *R*-factors(gt) etc. and is not relevant to the choice of reflections for refinement. *R*-factors based on *F*^2^ are statistically about twice as large as those based on *F*, and *R*- factors based on ALL data will be even larger.

### Fractional atomic coordinates and isotropic or equivalent isotropic displacement parameters (Å^2^)

**Table:**

	*x*	*y*	*z*	*U*_iso_*/*U*_eq_	Occ. (<1)
C1	0.15963 (5)	0.9135 (4)	0.2841 (2)	0.0424 (5)	
H1	0.1439	1.0293	0.2497	0.051*	
C2	0.19149 (5)	0.9476 (4)	0.2556 (3)	0.0470 (6)	
H2	0.1973	1.0855	0.2009	0.056*	
C3	0.21488 (5)	0.7785 (4)	0.3078 (3)	0.0484 (6)	
H3	0.2366	0.8022	0.2889	0.058*	
C4	0.20619 (5)	0.5744 (4)	0.3878 (3)	0.0457 (6)	
H4	0.2221	0.4604	0.4237	0.055*	
C5	0.17421 (5)	0.5374 (4)	0.4152 (2)	0.0403 (5)	
H5	0.1685	0.3980	0.4682	0.048*	
C6	0.15054 (5)	0.7074 (3)	0.3639 (2)	0.0352 (5)	
C7	0.11668 (5)	0.6746 (4)	0.4006 (2)	0.0370 (5)	
C8	0.09363 (5)	0.8478 (4)	0.4305 (3)	0.0438 (5)	
H8	0.0961	1.0098	0.4236	0.053*	
C9	0.06769 (5)	0.7318 (4)	0.4705 (3)	0.0460 (6)	
C10	0.03668 (5)	0.8061 (5)	0.5327 (3)	0.0629 (7)	
H10A	0.0377	0.7646	0.6615	0.076*	
H10B	0.0350	0.9757	0.5237	0.076*	
N1	0.10486 (4)	0.4641 (3)	0.4183 (2)	0.0501 (5)	
O1	0.07313 (3)	0.4982 (3)	0.4643 (2)	0.0543 (4)	
O2	0.00792 (4)	0.7069 (3)	0.4334 (3)	0.0808 (6)	
H2B	0.0046	0.5589	0.4396	0.121*	0.50
H2A	0.0167	0.6698	0.3350	0.121*	0.50

### Atomic displacement parameters (Å^2^)

**Table:**

	*U*^11^	*U*^22^	*U*^33^	*U*^12^	*U*^13^	*U*^23^
C1	0.0450 (13)	0.0391 (13)	0.0412 (10)	−0.0012 (10)	−0.0003 (9)	0.0019 (10)
C2	0.0546 (14)	0.0453 (14)	0.0412 (11)	−0.0104 (12)	0.0076 (9)	0.0033 (10)
C3	0.0405 (12)	0.0642 (17)	0.0415 (11)	−0.0082 (12)	0.0098 (9)	−0.0076 (11)
C4	0.0425 (13)	0.0513 (16)	0.0438 (11)	0.0086 (11)	0.0081 (9)	−0.0007 (11)
C5	0.0471 (12)	0.0362 (13)	0.0377 (10)	0.0027 (10)	0.0066 (8)	0.0018 (9)
C6	0.0393 (11)	0.0346 (12)	0.0308 (9)	−0.0001 (10)	0.0018 (7)	−0.0019 (8)
C7	0.0381 (11)	0.0332 (12)	0.0384 (10)	−0.0013 (10)	0.0015 (8)	−0.0001 (9)
C8	0.0378 (12)	0.0346 (13)	0.0571 (12)	0.0000 (11)	0.0006 (9)	−0.0002 (10)
C9	0.0384 (12)	0.0413 (14)	0.0562 (12)	0.0046 (11)	0.0001 (9)	−0.0029 (11)
C10	0.0400 (13)	0.0669 (19)	0.0812 (16)	0.0018 (13)	0.0068 (11)	−0.0049 (14)
N1	0.0402 (11)	0.0408 (12)	0.0702 (11)	0.0002 (9)	0.0112 (8)	0.0017 (9)
O1	0.0395 (9)	0.0458 (11)	0.0781 (10)	−0.0054 (8)	0.0105 (7)	0.0018 (8)
O2	0.0340 (9)	0.0818 (15)	0.1241 (15)	−0.0034 (9)	0.0039 (9)	−0.0057 (12)

### Geometric parameters (Å, °)

**Table:**

C1—C2	1.368 (3)	C7—N1	1.306 (3)
C1—C6	1.387 (3)	C7—C8	1.406 (3)
C1—H1	0.9300	C8—C9	1.322 (3)
C2—C3	1.373 (3)	C8—H8	0.9300
C2—H2	0.9300	C9—O1	1.351 (3)
C3—C4	1.373 (3)	C9—C10	1.476 (3)
C3—H3	0.9300	C10—O2	1.410 (3)
C4—C5	1.373 (3)	C10—H10A	0.9700
C4—H4	0.9300	C10—H10B	0.9700
C5—C6	1.383 (3)	N1—O1	1.406 (2)
C5—H5	0.9300	O2—H2B	0.8561
C6—C7	1.466 (3)	O2—H2A	0.8798
			
C2—C1—C6	120.5 (2)	N1—C7—C6	120.73 (18)
C2—C1—H1	119.7	C8—C7—C6	128.09 (19)
C6—C1—H1	119.7	C9—C8—C7	105.4 (2)
C1—C2—C3	120.1 (2)	C9—C8—H8	127.3
C1—C2—H2	119.9	C7—C8—H8	127.3
C3—C2—H2	119.9	C8—C9—O1	110.0 (2)
C2—C3—C4	119.9 (2)	C8—C9—C10	133.2 (2)
C2—C3—H3	120.1	O1—C9—C10	116.5 (2)
C4—C3—H3	120.1	O2—C10—C9	114.7 (2)
C5—C4—C3	120.4 (2)	O2—C10—H10A	108.6
C5—C4—H4	119.8	C9—C10—H10A	108.6
C3—C4—H4	119.8	O2—C10—H10B	108.6
C4—C5—C6	120.1 (2)	C9—C10—H10B	108.6
C4—C5—H5	120.0	H10A—C10—H10B	107.6
C6—C5—H5	120.0	C7—N1—O1	105.53 (16)
C5—C6—C1	119.00 (19)	C9—O1—N1	107.91 (15)
C5—C6—C7	120.49 (19)	C10—O2—H2B	119.6
C1—C6—C7	120.46 (18)	C10—O2—H2A	96.2
N1—C7—C8	111.11 (19)	H2B—O2—H2A	84.0
			
C6—C1—C2—C3	−0.7 (3)	N1—C7—C8—C9	1.2 (2)
C1—C2—C3—C4	0.3 (3)	C6—C7—C8—C9	−175.60 (17)
C2—C3—C4—C5	0.4 (3)	C7—C8—C9—O1	−0.9 (2)
C3—C4—C5—C6	−0.7 (3)	C7—C8—C9—C10	173.2 (2)
C4—C5—C6—C1	0.4 (3)	C8—C9—C10—O2	131.4 (3)
C4—C5—C6—C7	−177.04 (16)	O1—C9—C10—O2	−54.8 (3)
C2—C1—C6—C5	0.3 (3)	C8—C7—N1—O1	−1.0 (2)
C2—C1—C6—C7	177.73 (16)	C6—C7—N1—O1	176.11 (15)
C5—C6—C7—N1	−24.9 (3)	C8—C9—O1—N1	0.4 (2)
C1—C6—C7—N1	157.73 (18)	C10—C9—O1—N1	−174.81 (17)
C5—C6—C7—C8	151.63 (19)	C7—N1—O1—C9	0.4 (2)
C1—C6—C7—C8	−25.7 (3)		

### Hydrogen-bond geometry (Å, °)

**Table:**

*D*—H···*A*	*D*—H	H···*A*	*D*···*A*	*D*—H···*A*
O2—H2B···O2^i^	0.86	1.89	2.669 (4)	151
O2—H2A···O2^ii^	0.88	2.09	2.677 (5)	124
C8—H8···N1^iii^	0.93	2.61	3.542 (4)	177
C10—H10B···O2^iv^	0.97	2.58	3.352 (4)	137

Symmetry codes: (i) −*x*, −*y*+1, −*z*+1; (ii) −*x*, *y*, −*z*+1/2; (iii) *x*, *y*+1, *z*; (iv) −*x*, −*y*+2, −*z*+1.
